# The *Girl with a Pearl Earring*: A dermatological puzzle

**DOI:** 10.1111/jdv.20854

**Published:** 2025-07-14

**Authors:** Giampiero Girolomoni, Paolo Gisondi, Martina Maurelli

**Affiliations:** ^1^ Department of Medicine, Section of Dermatology University of Verona Verona Italy

**Keywords:** alopecia areata, Johannes Vermeer, madarosis, The *Girl with a Pearl Earring*

The *Girl with a Pearl Earring* (nicknamed the ‘*Mona Lisa of the North*’) is one of the most popular masterpieces in the world. It was painted by Johannes Vermeer ca.1665 (Figure 1).^1^, ^2^ The work has been in the collection of the Mauritshuis in The Hague since 1902 and has been the subject of various literary and cinematic treatments.^1^

**FIGURE 1:**
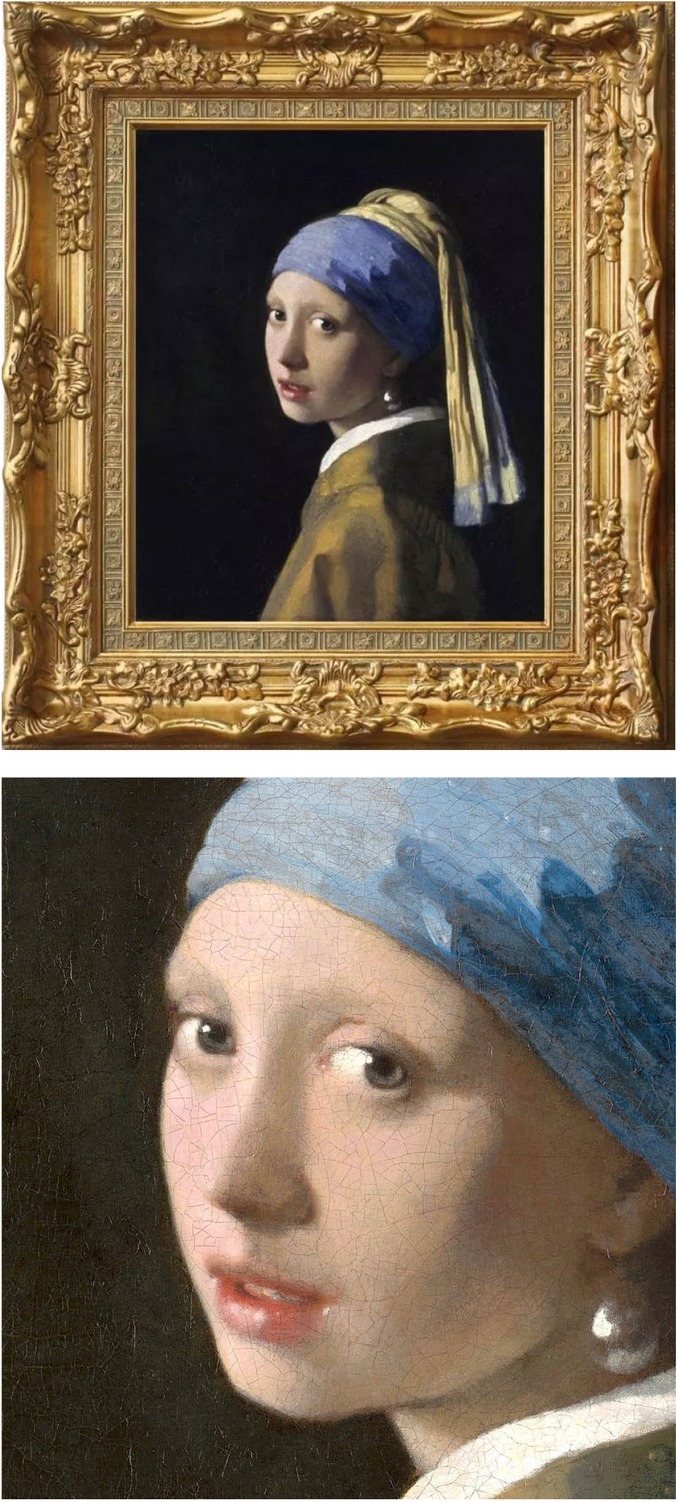
The *Girl with a Pearl Earring* by Johannes Vermeer (oil on canvas, 44.5 × 39 cm). Images downloaded from https://www.mauritshuis.nl.

Johannes Vermeer (Delft 1632–1675) is one of the foremost genre painters of the 17th century, depicting scenes from everyday life of ordinary people at work or at leisure. Fewer than 40 paintings are attributed to him, the most famous of which depict daily life in interior settings.^1^

The identity of the *Girl with a Pearl Earring* has been the subject of much speculation. Most experts believe that the portrait is not depicting a real identifiable girl, but rather it is ‘tronie’. Tronie translates from Dutch as ‘face’ or ‘expression’, and refers to a specific type of portrait, where a head or bust is depicted often with an exaggerated or characteristic facial expression, and sometimes in unusual or exotic costume. However, others have hypothesized that it is a portrait of the artist's eldest daughter, Maria, who would have been approximately 12 or 13 years old during the years 1665–1667.^1^, ^2^, ^3^, ^4^ Moreover, the painting has been extensively investigated to understand the materials and techniques used by Vermeer, using the most advanced imaging methods.^5^, ^6^

The *Girl with a Pearl Earring* is essentially a manifestation of beauty in simplicity. Neither the portrait nor the girl's face is particularly distinctive, but the delicate colour scheme against the black background and the intimacy of the girl's gaze toward the viewer with lips slightly parted is direct and captivating.

What is the interest of this painting for the dermatologist? Of particular interest for the fact that the girl appears at first glance to have no eyebrows or lashes, suggesting that she may have had alopecia areata. Also, the type of hat the girl wears is unusual for that period, a superb turban, perhaps worn because of alopecia of the scalp, rather than to convey exoticism. However, sparse eyelashes have been revealed by using macroscopic x‐ray fluorescence scanning,^6^ and thus, she may have had madarosis, a term indicating loss of the brows and lashes. Madarosis may have a variety of causes, including inflammatory (frontal fibrosing alopecia), infectious (syphilis), neoplastic (T cell lymphoma), metabolic (hypothyroidism) and genetic origin (keratosis follicularis spinulosa decalvans).^7^ The absence of erythema or any other skin sign as well as the age of the girl favours the hypothesis of alopecia areata. The possibility of syphilis has been mooted before.^8^ The lower lip has a small red area on the left side looking like an erosion, which has been suspected to be first‐stage chancre. However, alopecia is a manifestation of secondary syphilis and the red area on the lip might be the result of labial herpes or of an injury. Moreover, unlike areas with high iodine deficiency, goitre was less common in the Netherlands in the 17th century. Regardless of any medical considerations, the charm of the painting and of the girl is not diminished by the sparsity of hair. We are looking at a version of idealized beauty.

## FUNDING INFORMATION

None.

## CONFLICT OF INTEREST STATEMENT

None to disclose.

## ETHICAL APPROVAL

Not applicable.

## ETHICS STATEMENT

Not applicable.

## Data Availability

Not applicable.
